# Evaluating the Effectiveness of a Mobile App for Breast Cancer Self-Management on Self-Efficacy: Nonrandomized Intervention Trial

**DOI:** 10.2196/63989

**Published:** 2025-03-26

**Authors:** Sun Mi Kim, Da Seul Kim, Yoonsung Jang, Min Kyoon Kim, Eun-Seung Yu, Doug Hyun Han, Hee Jun Kim

**Affiliations:** 1 Department of Psychiatry College of Medicine Chung-Ang University Seoul Republic of Korea; 2 Department of Surgery College of Medicine Chung-Ang University Seoul Republic of Korea; 3 Department of Counseling Psychology The Cyber University of Korea Seoul Republic of Korea; 4 Division of Medical Oncology Department of Internal Medicine, College of Medicine Chung-Ang University Seoul Republic of Korea

**Keywords:** breast cancer, mobile health, mHealth, health education, self-efficacy, psychological adjustments, mobile phone

## Abstract

**Background:**

Numerous mobile apps have been developed for patients with cancer. However, there is still no comprehensive app for patients with breast cancer that integrates evidence-based medical information, psychological support, and schedule management through a multidisciplinary medical approach.

**Objective:**

We aimed to investigate whether a mobile app designed to assist in the self-management of patients with breast cancer is feasible and positively affects their self-efficacy and other psychological aspects.

**Methods:**

The Cancer Manager (CAMA) app was developed to assist in the self-management of patients with breast cancer and survivors of cancer according to cancer trajectory. Its functionalities include providing evidence-based digitalized information created by experts, managing patients’ medication and medical appointment schedules, and providing a delayed question and answer system for patients to query health care professionals. In this nonrandomized intervention trial, we analyzed data from 66 patients with breast cancer, divided into experimental (CAMA: n=34, 52%) and control (treatment as usual: n=32, 48%) groups. Group allocation was determined based on the patient’s willingness to use the app and access to compatible smartphones. Outcome measures included the Korean version of the Cancer Survivor Self-Efficacy Scale, the Korean version of the Mini-Mental Adjustment to Cancer (K-Mini-MAC) Scale, the World Health Organization Quality of Life Brief Version, Patient Health Questionnaire-9 (PHQ-9), Generalized Anxiety Disorder-7 (GAD-7), and Menopause Emotional Symptoms Questionnaire (MESQ). A user satisfaction survey was also conducted.

**Results:**

Throughout the intervention period, the CAMA group (vs treatment as usual group) demonstrated significant improvements in the seeking help and support subscale of the Korean version of the Cancer Survivor Self-Efficacy Scale (*F*_1,64_=5.09; *P*=.03), the psychological well-being subscale of the World Health Organization Quality of Life Brief Version (*F*_1,64_=5.48; *P*=.02), the anxious preoccupation subscale (*F*_1,64_=5.49; *P*=.02) and positive attitude subscale (*F*_1,64_=5.44; *P*=.02) of the K-Mini-MAC Scale, PHQ-9 (*F*_1,64_=4.83; *P*=.03), GAD-7 (*F*_1,64_=5.48; *P*=.02), and MESQ (*F*_1,64_=4.30; *P*=.04). Changes in the anxious preoccupation subscale of the K-Mini-MAC Scale scores were positively correlated with changes in the PHQ-9 (*r*=0.46; *P*=.007) and GAD-7 (*r*=0.41; *P*=.02) scores and negatively correlated with changes in the positive attitude subscale of the K-Mini-MAC Scale scores (*r*=–0.36; *P*=.04). Changes in the PHQ-9 scores were positively correlated with changes in the GAD-7 (*r*=0.66; *P*<.001) and MESQ (*r*=0.35; *P*=.04) scores. The user satisfaction survey offered insights into the CAMA app’s positive impact; trust-building outcomes; and opportunities for enhancement, such as the inclusion of communication tools and continued content enrichment.

**Conclusions:**

The mobile app for breast cancer self-management, CAMA, was deemed feasible and showed promise in improving the patients’ self-efficacy regarding seeking help and support, positive attitude toward cancer, and psychological well-being. In addition, its use might help reduce anxious preoccupation with cancer, depressive mood, anxiety, and menopausal emotional symptoms.

**Trial Registration:**

Clinical Research Information Service KCT0007917; https://cris.nih.go.kr/cris/search/detailSearch.do?seq=23348

## Introduction

### Background

Breast cancer is the most prevalent cancer among women worldwide, accounting for approximately 1 out of every 4 cancer diagnoses [1]. Despite advancements in breast cancer treatment, patients continue to experience significant psychological and physical burdens [2,3]. The wide range of subtypes of breast cancer adds to its complexity, as each has its distinct biological and genetic characteristics. This complexity leads to the need for highly individualized chemotherapy regimens tailored to factors such as hormone receptor status, human epidermal growth factor receptor 2 status, and genetic mutations [4,5]. Research also shows that patients with breast cancer often face, throughout the cancer journey, various unmet needs, particularly in areas such as physical and functional challenges, psychological support, and access to clear medical information [6-8]. More specifically, common issues include fatigue, pain, anxiety, fear of recurrence, difficulties in daily activity management, and a lack of coordinated care [9]. The needs of patients with breast cancer are also often more pronounced in younger patients and those with advanced cancer, especially during the transition from active treatment to survivorship [3,7].

Among patients with cancer, unmet needs have been linked to increased long-term distress and decreased quality of life (QOL) [10,11]. The symptom burden that arises from these unmet needs significantly influences the physical and psychological QOL of survivors of cancer [12]. Therefore, interventions to address these unmet needs and support the physical and psychological health of survivors of cancer are crucial at every stage of the cancer journey. However, in clinical settings, there can be physical constraints (eg, associated with staffing, space, and time limitations) to realize needs of all survivors of cancer, and attempts to address these needs may impose additional burdens on health care providers and systems. Given this reality, it is unsurprising that interest in using mobile apps in medical settings has been growing, enabling stakeholders to overcome challenges in addressing the needs of patients with cancer [13].

### Prior Work

Given that breast cancer is the most common cancer (vs other cancer types) among women with a higher prevalence of unmet needs, many cancer-related apps are primarily targeted at this population [14]. Mobile health (mHealth) apps for patients with breast cancer have also become increasingly sophisticated, integrating various functionalities to support patients throughout their treatment and survivorship phases. Indeed, the extant apps for this type of cancer address a range of needs, including information on survivorship, upper limb dysfunction, sleep disturbances, nutrition, general health education, emotional support, energy balance challenges, and motivation [15]. Furthermore, they facilitate continuous monitoring, real-time feedback, and resource provision, which are essential in managing the complexities of breast cancer treatment [15]. mHealth apps have been reported to provide significant benefits for symptom management, patient engagement, and self-efficacy, which in turn are all critical for successful long-term outcomes [16,17]. mHealth-based intervention implementation has also been shown to positively influence QOL, stress, and weight management of survivors of breast cancer [17]. Particularly, these interventions enable patients with breast cancer to report symptoms and treatment-related side effects, promoting effective self-management [18].

Despite the advances, current research on mHealth apps for patients with breast cancer features several gaps. For instance, most research-based apps focus on only 1 or 2 functions (eg, information delivery, adherence management, lifestyle enhancement, psychological support, or referral and linkage to resources) and thus fail to integrate all these features into a single app. In addition, most apps lack personalized recommendations based on individual patient data and face usability challenges, particularly for diverse populations [19,20]. Researchers have also posited that the effectiveness of the psychological support features of mHealth apps is subpar and that the specific needs of different breast cancer subtypes are underexplored [21]. Studies have traditionally focused on improving QOL [22], often at the expense of investigating other critical clinical outcomes and the cost-effectiveness of these technologies in health care settings [23].

### Theory

The development of our app, especially its structure and functionality, was guided by 2 pivotal theoretical frameworks, namely, the chronic care model (CCM) and the health belief model (HBM). The CCM emphasizes the importance of an integrated approach to managing chronic diseases such as breast cancer [24,25]. It advocates for a patient-centered approach that not only focuses on treating the disease but also on improving patients’ self-management capabilities and fostering long-term wellness [25]. The CCM informed our app’s design by its emphasis on continuous and integrative care that incorporates medical, psychological, and social support. For example, features such as real-time symptom tracking, personalized health education, and coordinated care planning were developed to align with the CCM’s core elements, ensuring that the app supports patients in managing their health proactively and holistically [26,27]. Meanwhile, the HBM was essential in addressing the psychological and behavioral aspects of health management within the app. The HBM posits that individuals’ health-related behaviors are influenced by their perceptions of the severity of a health condition, their susceptibility to the condition, the benefits of taking preventive action, and the barriers to doing so [28]. This model was particularly influential in the development of the app’s psychological support module, which aims to enhance patients’ motivation and confidence in condition management [29]. By incorporating tools that address perceived barriers (eg, educational resource provision) and highlighting the benefits of adherence to treatment and self-care routines, the app was designed to empower patients to take an active role in managing their health [30,31].

These frameworks provided a structured approach for the development of the app, ensuring that it not only meets the immediate medical needs but also supports long-term behavioral change and self-efficacy improvement among patients with breast cancer. The integration of CCM and HBM into the app enabled the delivery of proactive and personalized patient-centered care that is supportive of the complex psychological and physical needs of survivors of breast cancer.

### Study Aim

This study aimed to develop a self-management support app tailored to the needs of patients with breast cancer and verify its feasibility. Furthermore, we investigated whether the app would have a positive effect on patient self-efficacy and other psychological aspects compared to a control group—hereinafter treatment as usual (TAU) group—that did not use the app. In this study, we chose self-efficacy as the primary outcome based on the self-efficacy theory by Bandura [32], which posits that individuals with higher self-efficacy are more likely to engage in self-management behaviors, leading to better health outcomes [33]. This theory is particularly relevant in chronic disease management, where patient engagement in treatment and self-care practices is crucial for long-term treatment success. Enhancing self-efficacy can empower patients to better cope with the physical and psychological burdens associated with breast cancer, thus improving their overall QOL.

Our research differs from previous work because it involved the incorporation of a broader range of features into a single app, providing a more holistic and culturally sensitive tool (vs other similar apps) tailored to the specific needs of patients with breast cancer. By addressing the identified shortcomings in the literature and using a multidisciplinary approach, we aimed to offer a comprehensive mHealth solution where other similar apps have focused on fewer aspects of care. Consequently, we developed 4 modules: an evidence-based medical information module, a psychological support module, a medication and appointment management module, and a patient inquiry module. On the basis of these aims, we proposed the following hypotheses:

- Hypothesis 1: app use will significantly improve self-efficacy among patients with breast cancer compared to routine care.
- Hypothesis 2: app use will significantly improve QOL; mental adjustment to cancer; and psychological symptoms such as depression, anxiety, and menopausal emotional symptoms.
- Hypothesis 3: there will be a statistically significant association between the amount of change in the psychological variables that improved after app use.

## Methods

### Study Design

This study was a 12-week nonrandomized controlled intervention trial. This study used a nonrandomized design, selected mainly for convenience and logistical considerations. Randomized group assignment was deemed infeasible within the scope of this study due to resource and time constraints, as implementing randomization would have required additional infrastructure and staffing. While this design choice imposed certain limitations, it allowed for efficient recruitment and management of participants in real-world clinical settings. This study was reported in accordance with the TREND (Transparent Reporting of Evaluations with Nonrandomized Designs) statement, and a completed TREND checklist is provided in Multimedia Appendix 1.

### Ethical Considerations

The study protocol was reviewed and approved by the Institutional Review Board of Chung-Ang University Hospital (2201-025-494). All participants provided written informed consent before enrollment in the study. Participant data were anonymized and stored on a secure server accessible only to the research team. Participants received approximately US $14 as compensation for each of their baseline and follow-up visits. The clinical trial registration was approved by the Clinical Research Information Service (KCT0007917).

### Study Participants and Recruitment

Using convenience sampling, 80 patients with pathologically proven clinical stage 0 to 4 breast cancer were recruited through an advertisement in hospital bulletins of the Digital Cancer Center at Chung-Ang University Hospital, located in Seoul, Korea, from January 2023 to June 2024. We focused on patients who voluntarily indicated their interest in participating in the study, and those who agreed to participate in the study were screened according to the following inclusion criteria: an adult female who was either undergoing active treatment for breast cancer or was a survivor of breast cancer. The exclusion criteria were as follows: current or history of uncontrolled medical diseases other than cancer; current or history of uncontrolled psychiatric diagnosis, including severe mood disorders, alcohol or substance use disorders, and intellectual disabilities; inability to engage in linguistic communication; and inability to use mobile apps on the phone.

Participants were assigned to the Cancer Manager (CAMA) group (ie, the experimental group) if they indicated a willingness to use the app and had access to a compatible smartphone, while those who did not meet these criteria were assigned to the TAU group. CAMA group participants used a mobile app, CAMA (HuDIT), designed to assist in the self-management of patients with breast cancer, and received routine care and education in the hospital; TAU group participants only received routine care and education. Routine care for patients with cancer aims to deliver comprehensive health care management services; does not encompass additional digital interventions; and typically includes regular medical follow-ups, symptom management, psychological support, nutrition counseling, palliative care, patient education, care coordination, monitoring and testing, access to emergency care, and social support services.

The eligibility screening and informed consent procedures were conducted by physicians (SMK, MKK, or HJK) or researchers (DSK or YJ). After the researchers provided explanations about the app, the app was installed on the patient’s device either by the researchers or by the patients themselves. Then, the researchers provided participants with appropriate instructions about the intervention, including how to use the app, the assessment schedule, and how to contact the *Manager* of the CAMA or research team. Patients were instructed to access the app daily and use it for at least 10 minutes. We administered the baseline and follow-up questionnaires from January 2023 to June 2024 via in-person visits or phone calls.

**Figure 1:**
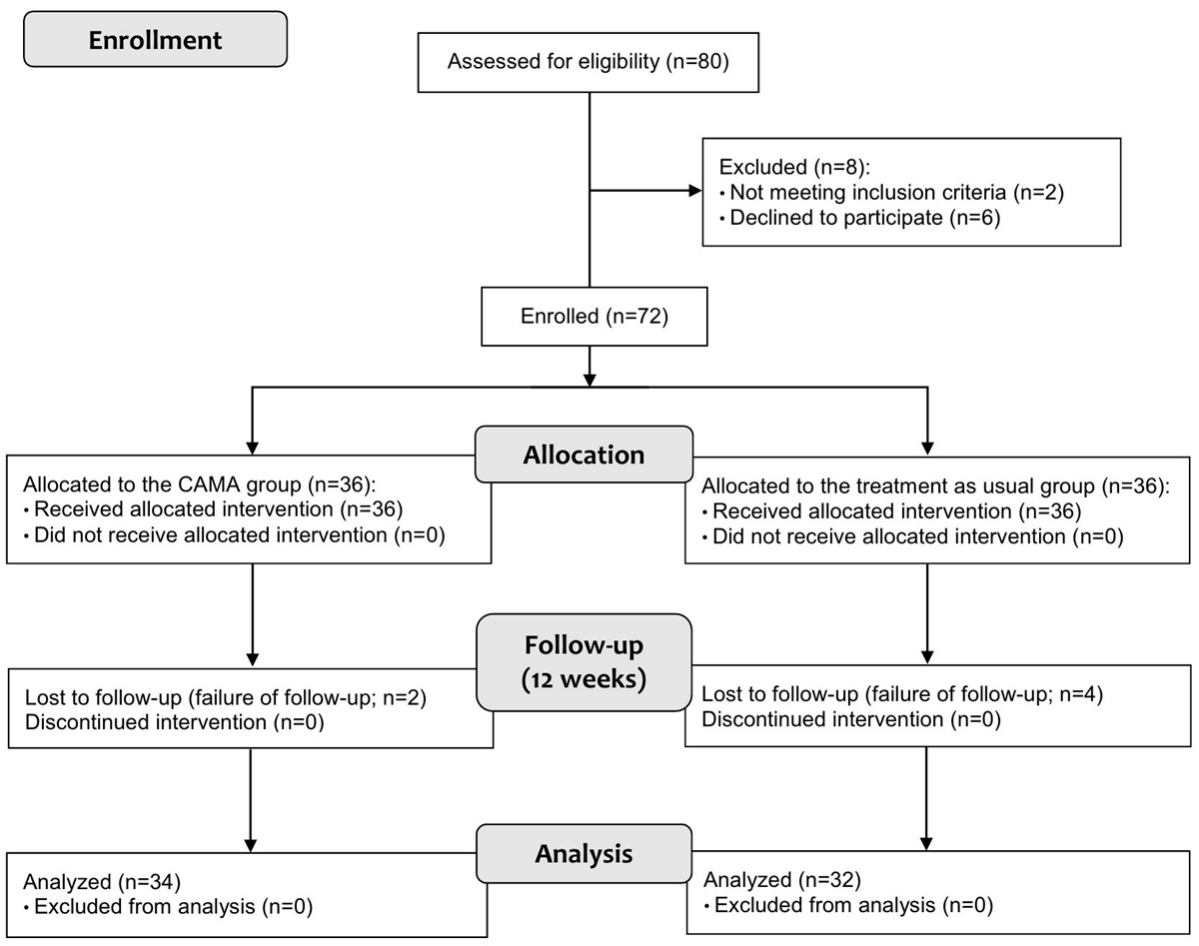
Study flow diagram. CAMA: Cancer Manager.

In total, 80 patients with breast cancer underwent eligibility assessment (Figure 1), and some were excluded because of the following reasons: 6 (8%) declined to participate before enrollment, 1 (1%) had a major depressive disorder with agitation and suicidal ideation, and 1 (1%) was diagnosed with major neurocognitive disorder due to Alzheimer disease.

### Allocation

We enrolled 72 participants based on the inclusion and exclusion criteria, with 36 (50%) participants each in the experimental (CAMA) and TAU groups (Figure 1). As aforementioned, group allocation was determined based on patients’ willingness to use the app and their access to compatible smartphones. This study did not use matching techniques for the nonrandomized assignment procedures. The CAMA group used the CAMA mobile app for 12 weeks, whereas the TAU group received no intervention during that period. Both groups maintained their usual treatment regimens throughout the 12 weeks. Clinical assessments of self-efficacy and other psychological aspects were conducted immediately after the beginning of the experiment (eg, right before patients downloaded the app and started using it) and immediately after the end of the experiment (eg, 1 day after the formal ending of the experiment).

### App Development: CAMA

#### Overview

The CAMA app was developed to support patients with breast cancer in their self-management across different phases of the cancer journey, including at the time of diagnosis, during active treatment, and after treatment completion (ie, during the cancer survivorship phase). The CAMA app had three main functionalities: (1) it provided evidence-based digitalized information created by experts, (2) it managed patients’ medication and medical appointment schedules, and (3) it delivered a delayed question and answer (Q and A) system whereby patients can ask questions to health care professionals.

#### Evidence-Based Digitalized Information

**Figure 2:**
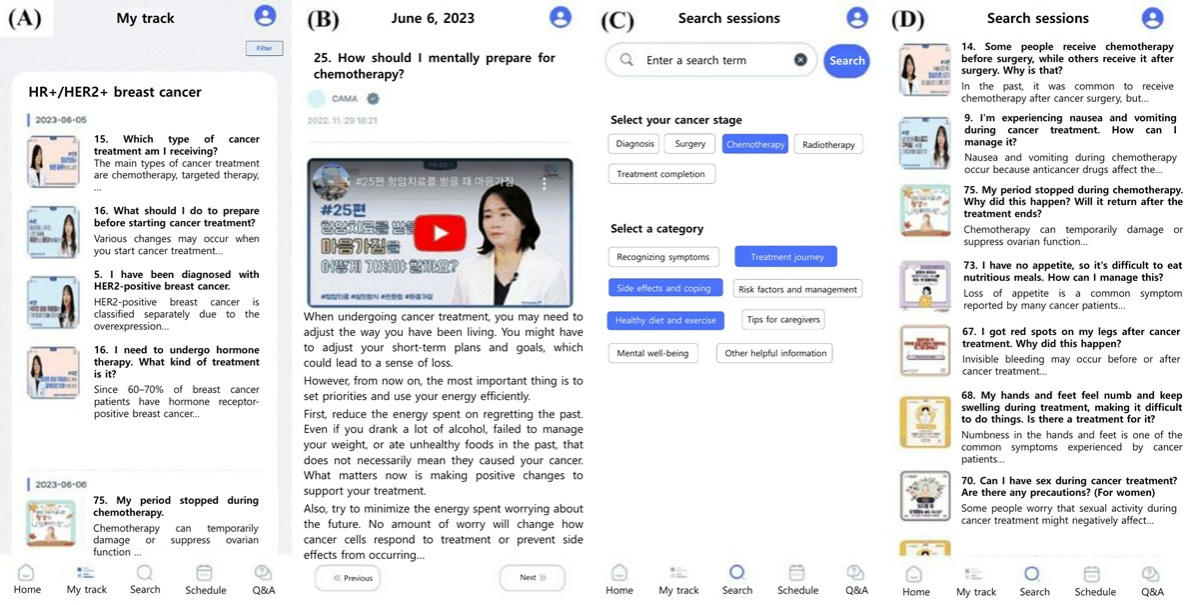
Representative screens of the My care track and information content search features of the Cancer Manager app. This figure was created by translating the original Korean text from the app's screenshots into English. (A) My care track feature: this function allows patients to receive personalized information relevant to their breast cancer type (eg, hormone-positive and human epidermal growth factor receptor 2-positive) and treatment phase (eg, chemotherapy) through daily alarms. (B) Information content: clicking on the alarm window provides information to read on that day in the form of videos, infographics, and articles. All information was authored by experts in their respective fields (eg, hematologic oncology, breast surgery, psychiatry, and psychology). (C) Information content search feature: this function allows patients to directly search for the information they need. They can either enter search terms directly through the search box (at the top) or click on keywords for specific cancer trajectories and areas (at the bottom) before clicking the search button. (D) Information content search results: after entering search terms or clicking on keywords, pressing the search button displays a list of relevant contents, and a subsequent click on an item on the list reveals its details.

First, oncologists, surgeons, psychiatrists, and psychologists were recruited to create informative content tailored to each phase of the cancer journey (Figure 2). These digitalized contents, including videos, articles, images, and infographics, were registered on the website of the administrator of the CAMA app. The CAMA app provides personalized information to the patient depending on whether one is undergoing chemotherapy, surgery, or radiation therapy or is in the cancer survivorship phase. The digital contents covered topics such as information about breast cancer diagnosis, symptom management, preparation for treatment, management of treatment side effects, diet and nutrition, rehabilitation and exercise, psychological support, and information for caregivers.

Physicians and a nurse, a social worker, or a psychologist (referred to as the *Manager*) define the most relevant content according to the cancer-related condition (referred to as *My Care Track*), and the *Manager* registers the content on the administrator’s website. Alternatively, patients can search for and access information based on their requirements.

The *My Care Track* provides tailored content on a patient-specific basis, delivering helpful information regarding the patient’s cancer treatment stage. The contents are listed by date, and the app sends reminders and provides recommended content daily. For instance, the app considers factors such as the patient’s breast cancer subtype, time since cancer diagnosis, ongoing chemotherapy, or upcoming radiation therapy, providing essential daily guidance in concise, manageable pieces.

The *Manager* (referring to the nurse, social worker, or psychologist here) of the CAMA app is also responsible for coordinating the interactions between physicians and patients and the app and the offline treatment process. On the basis of previous research findings, barriers to the effectiveness of health care delivery through apps include a lack of feedback and support, a perception of the app as impersonal and cold, excessive or complicated information or intervention, discrepancies between the current treatment status and the information delivered, insufficient understanding of program use methods, and a lack of motivation [34]. The *Manager* of the CAMA app served to overcome these challenges, providing feedback and support, serving as a human communication channel, delivering tailored information or interventions, offering technical support for app use, and reinforcing motivation for app use. The *Manager* provided a communication channel between patients and health care professionals that was available during working hours (9 AM to 5 PM, Monday to Friday). In addition, every 2 weeks, the *Manager* texted participants to encourage the consistent use of the app. Every 4 weeks, the *Manager* called participants to check their frequency of use and how many days per week they used the app. Throughout the study, participants in the CAMA group interacted with health care professionals via the app an average of 0.44 (SD 0.93) times, with 15 interactions observed among the 34 participants in the CAMA group. Among the participants who completed the study, all met the minimum requirement of using the app for at least 10 minutes daily, resulting in a 100% engagement rate among these participants throughout the study procedures. Of 34 participants, 2 (6%) in the CAMA group who failed to meet this requirement or withdrew from the study were not included in the final analysis.

#### Patients’ Medication and Appointment Schedules

**Figure 3:**
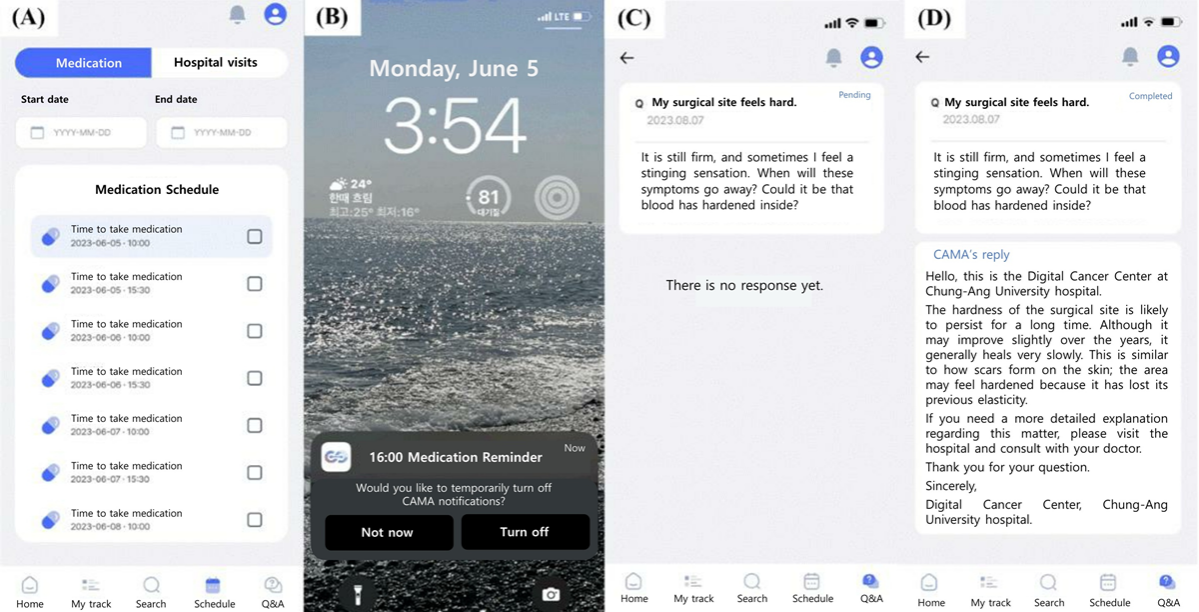
Representative screens for the medication and appointment reminder and delayed question and answer system features of the Cancer Manager app. This figure was created by translating the original Korean text from the app's screenshots into English. (A) and (B) Medication and appointment reminder features: this feature provides alarms for individual medication and medical appointment schedules, enabling patients to mark them as completed and supporting treatment adherence and monitoring. (C) and (D) Delayed question and answer system feature: patients can post questions on the app regarding cancer-related issues about which they are curious. After the Manager gathers these questions, they are forwarded to a relevant professional (eg, a designated physician or psychologist) to provide answers accordingly, who sends the answers back to the Manager within a few days. Subsequently, the Manager organizes the information and responds to the patients through the app.

In the CAMA app, the *Manager* can register the patient’s medication and medical appointment schedules through the website, or the patient can directly input these pieces of information into the app (Figure 3). An alert is triggered when the scheduled time arrives, and once the patient completes their medication or appointment, they can mark it as completed in the app.

#### Delayed Q and A System

Patients can choose whether they would like to have face-to-face or remote consultations with the *Manager* and can use the app to ask questions to health care professionals, including attending physicians. When a patient asks a question, the health care professionals must review it and respond within a few days. This delayed Q and A system—not a live one—was introduced to alleviate the burden on health care professionals and ensure the sustainability of the app. Throughout the study, an average of 1.41 questions were asked per participant in the CAMA group through the delayed Q and A system (48 times among the 34 CAMA group participants).

### Data Collection

#### Sociodemographic and Clinical Characteristics

This study collected data on the following: sociodemographic characteristics (eg, age, years of education, and monthly family income); clinical characteristics, including cancer stage at enrollment, whether initial or recurring cancer at enrollment, performance status, pathological characteristics, receptor expression status (eg, hormone receptors such as estrogen receptor or progesterone receptor and human epidermal growth factor receptor 2), and triple-negative phenotype; cancer treatment types being received (eg, chemotherapy, surgery, and radiation therapy); and whether the patient is in the cancer survivorship phase.

#### Psychological Characteristics and User Satisfaction

Participants’ self-efficacy, QOL, mental adjustment to cancer, and psychological symptoms (ie, depression and anxiety) were assessed using validated scales at baseline and after the 12-week intervention. A satisfaction survey was also conducted among the participants who completed 12 weeks of CAMA use.

#### Measurements

The Eastern Cooperative Oncology Group scale [35] was used to assess patient performance status based on physical activity. It was measured by health care providers and comprised 5 grades ranging from 0 (no symptoms) to 4 (completely bedridden throughout the day).

To evaluate self-efficacy, we used the 10-item Korean version of the Cancer Survivor Self-Efficacy Scale (CS-SES-K) [36,37]. This self-reported tool comprises 2 subscales and 10 items, namely, managing health problems (5 items) and seeking help and support (5 items). The items were rated on a scale ranging from 1 (not at all confident) to 10 (totally confident), and scores were calculated by adding the scores for each item, with higher scores indicating greater self-efficacy. The CS-SES-K demonstrated high reliability and validity when applied to Korean cancer survivors [37], and the Cronbach α was 0.92 for the original tool [36] and the Korean version [37]. In our study, the internal consistency of the CS-SES-K was also high, with a Cronbach α of 0.93.

To assess mental adjustment to cancer, we used the 29-item Korean version of the Mini-Mental Adjustment to Cancer (K-Mini-MAC) Scale. This 29-item self-reported tool categorizes cognitive and behavioral responses to cancer into 4 types: helplessness or hopelessness (8 items), anxious preoccupation (8 items), cognitive avoidance (4 items), and positive attitude (fatalism and fighting spirit; 9 items) [38,39]. Items were rated on a scale ranging from 1 (definitely does not apply to me) to 4 (definitely applies to me), with higher scores indicating a more pronounced inclination to exhibit a specific response. This scale demonstrated acceptable reliability and validity when applied to Korean survivors of cancer [38]. The Cronbach α range was 0.62 to 0.88 for the original tool [39] and 0.72 to 0.86 for the Korean version [38]. In our study, the Cronbach α ranged from 0.74 to 0.89.

To evaluate QOL, we used the World Health Organization Quality of Life Brief Version (WHOQOL-BREF) [40,41]. This 26-item self-reported tool comprises 2 items measuring overall QOL and general health, along with 24 items divided into 4 subscales: physical health (7 items), psychological well-being (6 items), social relationships (3 items), and environmental aspects (8 items). Each item was scored on a scale ranging from 1 (not at all or very dissatisfied) to 5 (completely or very satisfied) with reverse-scoring items. Scoring was based on a specific formula, and higher scores on each subscale indicated a higher QOL in that respective domain [41]. This scale has demonstrated good validity, internal consistency, and test-retest reliability across various populations [40,41]. The Cronbach α range was 0.66 to 0.82 for the original tool [40] and 0.90 for the Korean version [41]. In this study, the Cronbach α range was 0.67 to 0.84, indicating high internal consistency.

The 9-item Patient Health Questionnaire-9 (PHQ-9) was used to assess depression and depressive symptom severity [42,43]. This self-reported tool was responded to on a 4-point Likert scale ranging from 0 (none) to 3 (almost every day), and each item assessed how often a responder had experienced depressive symptoms in the past 2 weeks. The scores were calculated by adding the scores for each item, with higher scores indicating more severe depression. A previous study conducted with Korean psychiatric outpatients showed an internal consistency reliability of 0.81 and a test-retest reliability of 0.89, with significant concurrent validity [43]. Moreover, the Cronbach α was 0.89 for the original tool [42] and 0.84 in this study.

To assess the degree of anxiety and anxiety symptoms, we used the 7-item Generalized Anxiety Disorder-7 (GAD-7) [44,45]. This self-reported tool was responded to on a 4-point Likert scale ranging from 0 (none) to 3 (almost every day), and each item assessed how often one had experienced anxiety symptoms in the past 2 weeks. The scores were calculated by adding the scores for each item, with higher scores indicating more severe anxiety. The GAD-7 demonstrated high validity and reliability for assessing anxiety in Korean psychiatric outpatients [45]. The Cronbach α was 0.92 for the original tool [44] and 0.88 for the Korean version [45]. In this study, Cronbach α was 0.90, demonstrating high internal consistency.

The Menopause Emotional Symptoms Questionnaire (MESQ) measures the emotional symptoms experienced by menopausal women. This self-reported scale comprises 13 items rated on a 5-point scale ranging from 0 (never experienced) to 4 (experienced very frequently). The MESQ is a valid and reliable tool for screening and measuring emotional symptoms in Korean menopausal women [46]. This scale was included because many patients with breast cancer had hormone-positive breast cancer and experienced menopause-like feelings while undergoing antihormonal therapy. In this study, the Cronbach α of 0.94 suggested a high internal consistency for the scale.

To assess satisfaction with CAMA use, we created a 17-item satisfaction questionnaire based on questionnaires used in previous studies [47]. Each self-reported item was rated on a 5-point Likert scale ranging from 0 (strongly disagree) to 5 (strongly agree). An additional open-ended question was included for responders to provide comments to app developers and the medical team.

### Sample Size Calculation

The target sample size was calculated using G*Power (version 3.1.9.7; Faul, Universität Kiel). A Wilcoxon signed rank test was considered necessary to compare the impact of CAMA use in a group of survivors of cancer. At the time of this study, no previous research had used the CS-SES-K as the primary outcome. Considering these descriptions and based on the effect size in a previous study evaluating the impact of a digital intervention on the self-efficacy of patients with cancer [48], a significance level of 0.05, power of 0.85, and an effect size of 0.20 (medium), the sample size required was 60 participants. Given a 15% dropout rate, the goal was to recruit 72 participants, with 36 (50%) participants in each group, a number this study could reach.

### Statistical Analyses

All statistical analyses were performed using SPSS for Windows (version 28.0; IBM Corp). The analysis was restricted to data from participants who had no missing values and completed both the pre- and postintervention assessments, which means that a per-protocol analysis approach was used. Independent samples 2-tailed *t* tests and chi-square tests were used to compare baseline characteristics between the CAMA and TAU groups. To evaluate the effectiveness of the CAMA app on primary and secondary outcomes, a mixed-effects ANOVA was conducted, with group (CAMA vs TAU) as a between-subject factor and time (baseline vs follow-up) as a within-subject factor. The primary outcome was self-efficacy (CS-SES-K scores), while the secondary outcomes included psychological well-being (WHOQOL-BREF scores), mental adjustment to cancer (K-Mini-MAC Scale scores), depressive symptoms (PHQ-9 scores), anxiety (GAD-7 scores), and menopausal emotional symptoms (MESQ scores). Pearson correlation analyses were conducted within the CAMA group to explore the relationships between changes in psychological outcomes. Moreover, we performed Pearson correlation analyses for scale scores that demonstrated a statistically significant improvement from baseline to follow-up.

## Results

### Participant Allocation and Baseline Characteristics

As mentioned in the *Methods* section, 80 patients with breast cancer underwent an eligibility assessment (Figure 1), but only 72 (90%) were enrolled, and 66 (82%) completed the study (completion rate: 66/72, 92%). In the CAMA group, discontinuation resulted from noncompliance with CAMA use in 2 participants; in the TAU group, 4 patients did not undergo any follow-up assessments (discontinuation rates: 2/36, 6% in the CAMA group and 4/36, 11% in the TAU group). Throughout the study period, no adverse events associated with the CAMA intervention were observed.

Results of the independent samples 2-tailed *t* test and chi-square test indicated no significant differences in the demographic factors between the CAMA and TAU groups at baseline (Table 1). Similarly, the CAMA and TAU groups exhibited no significant differences at baseline in the subscale and total scores for the CS-SES-K; the subscale scores for the WHOQOL-BREF and K-Mini-MAC Scale; and the total scores for the PHQ-9, GAD-7, and MESQ (Table 1).

**Table 1:** Sociodemographic and clinical characteristics at baseline.

Variables				CAMA^a^ group (n=34)		TAU^b^ group (n=32)		*t* test (*df*=64)			χ^2^ (*df*)			*P* value	
**Sociodemographic variables**															
	Age (y), mean (SD)		55.2 (7.8)		52.3 (8.8)		1.41			—^c^			.16		
	Years of education, mean (SD)		13.5 (2.3)		14.0 (2.9)		–0.73			—			.47		
	**Living with a partner, n (%)**							—			0.51 (21)			.48	
		Yes	27 (79)		23 (72)										
		No	7 (21)		9 (28)										
	Monthly family income (USD), mean (SD)		3329.0 (3851.5)		4686.1 (6735.0)		–1.01			—			.32		
	**Job, n (%)**							—			0.06 (21)			.80	
		Yes	17 (50)		17 (53)										
		No	17 (50)		15 (47)										
	**Religion, n (%)**							—			2.22 (21)			.14	
		Yes	26 (76)		19 (59)										
		No	8 (24)		13 (41)										
**Clinical characteristics**															
	Duration from breast cancer diagnosis (mo), mean (SD)		40.7 (68.9)		38.3 (47.4)		0.17			—			.87		
	**Performance status (ECOG^d^), n (%)**							—			0.91 (21)			.34	
		0	10 (29)		13 (41)										
		1	24 (71)		19 (59)										
	**Histology, n (%)**						—			0.50 (21)			.48		
		Invasive ductal carcinoma	21 (62)		17 (53)										
		Others	13 (38)		15 (47)										
	**ER^e^, n (%)**							—			0.91 (21)			.34	
		Negative	10 (29)		13 (41)										
		Positive	24 (71)		19 (59)										
	**PR^f^, n (%)**							—			2.15 (21)			.14	
		Negative	13 (38)		18 (56)										
		Positive	21 (62)		14 (44)										
	**HER2^g^, n (%)**							—			<0.001 (21)			.99	
		Negative	18 (53)		17 (53)										
		Positive	16 (47)		15 (47)										
	**Triple-negative phenotype, n (%)**							—			0.53 (21)			.47	
		Nontriple negative	28 (82)		24 (75)										
		Triple negative	6 (18)		8 (25)										
	**Cancer stage at enrollment, n (%)**							—			3.12 (24)			.54	
		0	4 (12)		7 (22)										
		1	3 (9)		5 (16)										
		2	7 (21)		3 (9)										
		3	1 (3)		1 (3)										
		4	19 (56)		16 (50)										
	**Cancer recurrence, n (%)**							—			0.53 (21)			.47	
		Initial	28 (82)		24 (75)										
		Relapse	6 (18)		8 (25)										
	**Metastatic location, n (%)**							—			1.39 (22)			.50	
		No metastasis	18 (53)		21 (66)										
		Nonvisceral lesion	10 (29)		8 (25)										
		Visceral lesion	6 (18)		3 (9)										
**Treatment phase at enrollment, n (%)**									—			3.94 (24)			.27
		Diagnosis	0 (0)		0 (0)										
		Surgery	1 (3)		5 (16)										
		Chemotherapy	26 (76)		23 (72)										
		Radiotherapy	3 (9)		1 (3)										
		Survivor	4 (12)		3 (9)										

^a^CAMA: Cancer Manager.

^b^TAU: treatment as usual.

^c^Not applicable.

^d^ECOG: Eastern Cooperative Oncology Group.

^e^ER: estrogen receptor.

^f^PR: progesterone receptor.

^g^HER2: human epidermal growth factor receptor 2.

### Comparison of Changes in Clinical Scale Scores Between the CAMA and TAU Groups

Throughout the intervention period, the CAMA group (vs the TAU group) demonstrated observable improvements in the following scale scores: the seeking help and support subscale of CS-SES-K (*F*_1,64_=5.09; *P*=.03), the psychological well-being subscale of WHOQOL-BREF (*F*_1,64_=5.48; *P*=.02), the anxious preoccupation subscale (*F*_1,64_=5.49; *P*=.02) and the positive attitude subscale (*F*_1,64_=5.44; *P*=.02; Table 2) of the K-Mini-MAC Scale, PHQ-9 (*F*_1,64_=4.83; *P*=.03), GAD-7 (*F*_1,64_=5.48; *P*=.02), and MESQ (*F*_1,64_=4.30; *P*=.04; Table 3). No significant differences were observed between the 2 groups in total or subscale scores for the other scales.

**Table 2:** Differences in the mean scores for the self-efficacy and psychological scales at baseline.

Psychological scales and variables			CAMA^a^ group (n=34), mean (SD)		TAU^b^ group (n=32), mean (SD)		*t* test (*df*=64)		*P* value
**CS-SES-K^c^**									
	Managing health problems	33.4 (10.2)		32.7 (11.8)		0.23		.82	
	Seeking help and support	38.2 (7.8)		37.2 (9.8)		0.47		.64	
	Total	71.6 (16.9)		69.9 (20.2)		0.36		.72	
**WHOQOL-BREF^d^**									
	Physical health	12.6 (3.1)		13.4 (2.0)		–1.26		.21	
	Psychological well-being	13.1 (3.7)		14.0 (3.4)		–1.04		.30	
	Social relationships	13.9 (2.7)		14.0 (2.6)		–0.09		.93	
	Environmental aspects	13.4 (2.8)		14.6 (2.8)		–1.79		.08	
**K-Mini-MAC^e^ Scale**									
	Helplessness or hopelessness	13.4 (4.6)		13.2 (5.4)		0.18		.86	
	Anxious preoccupation	22.2 (5.0)		20.4 (5.8)		1.36		.18	
	Cognitive avoidance	11.1 (2.6)		10.1 (2.9)		1.61		.11	
	Positive attitude (fatalism and fighting spirit)	27.3 (4.7)		27.2 (7.2)		0.09		.93	
PHQ-9^f^			8.3 (4.3)		7.0 (5.4)		1.09		.28
GAD-7^g^			6.7 (4.4)		4.7 (4.0)		1.91		.06
MESQ^h^			23.7 (10.4)		19.5 (9.8)		1.69		.10

^a^CAMA: Cancer Manager.

^b^TAU: treatment as usual.

^c^CS-SES-K: Korean version of the Cancer Survivor Self-Efficacy Scale.

^d^WHOQOL-BREF: World Health Organization Quality of Life Brief Version.

^e^K-Mini-MAC: Korean version of the Mini-Mental Adjustment to Cancer.

^f^PHQ-9: Patient Health Questionnaire-9.

^g^GAD-7: Generalized Anxiety Disorder-7.

^h^MESQ: Menopause Emotional Symptoms Scale.

**Table 3:** Comparison of score changes in the self-efficacy and psychological scales between the Cancer Manager (CAMA) and treatment as usual (TAU) groups.

Variables			CAMA group (n=34), mean (SD)					TAU group (n=32), mean (SD)					Group × time effect (mixed ANOVA)				
			Baseline		Follow-up		Baseline			Follow-up		*F* test (*df*=1,64)			*P* value		Partial η^2^
**CS-SES-K^a^**																	
	Managing health problems	33.4 (10.2)		33.1 (9.9)		32.7 (11.8)			29.8 (11.0)		1.15			.29		0.018	
	Seeking help and support	38.2 (7.8)		39.9 (6.9)		37.2 (9.8)			34.5 (10.6)		5.09			.03		0.074	
	Total	71.6 (16.9)		72.9 (15.2)		69.9 (20.2)			64.3 (20.2)		3.23			.08		0.048	
**WHOQOL-BREF^b^**																	
	Physical health	12.6 (3.1)		12.0 (2.8)		13.4 (2.0)			12.6 (3.2)		0.03			.86		<0.001	
	Psychological well-being	13.1 (3.7)		13.8 (2.7)		14.0 (3.4)			12.7 (3.6)		5.48			.02		0.079	
	Social relationships	13.9 (2.7)		13.3 (2.4)		14.0 (2.6)			12.6 (3.5)		1.56			.22		0.024	
	Environmental aspects	13.4 (2.8)		13.8 (2.4)		14.6 (2.8)			14.0 (2.5)		2.32			.13		0.035	
**K-Mini-MAC^c^ Scale**																	
	Helplessness or hopelessness	13.4 (4.6)		13.3 (4.6)		13.2 (5.4)			13.5 (4.6)		0.17			.68		0.003	
	Anxious preoccupation	22.2 (5.0)		19.2 (4.8)		20.4 (5.8)			19.9 (5.3)		5.49			.02		0.079	
	Cognitive avoidance	11.1 (2.6)		11.1 (2.6)		10.1 (2.9)			10.2 (2.7)		0.12			.73		0.002	
	Positive attitude (fatalism and fighting spirit)	27.3 (4.7)		27.8 (4.3)		27.2 (7.2)			25.3 (5.3)		5.44			.02		0.078	
PHQ-9^d^			8.3 (4.3)		6.6 (4.0)		7.0 (5.4)			8.0 (6.4)		4.83			.03		0.070
GAD-7^e^			6.7 (4.4)		5.5 (4.4)		4.7 (4.0)			5.7 (5.0)		5.48			.02		0.079
MESQ^f^			23.7 (10.4)		17.4 (9.4)		19.5 (9.8)			19.3 (12.7)		4.30			.04		0.063

^a^CS-SES-K: Korean version of the Cancer Survivor Self-Efficacy Scale.

^b^WHOQOL-BREF: World Health Organization Quality of Life Brief Version.

^c^K-Mini-MAC: Korean version of the Mini-Mental Adjustment to Cancer.

^d^PHQ-9: Patient Health Questionnaire-9.

^e^GAD-7: Generalized Anxiety Disorder-7.

^f^MESQ: Menopause Emotional Symptoms Questionnaire.

### Correlations Among Score Changes in Self-Efficacy and Psychological Scales Within the CAMA Group

**Figure 4:**
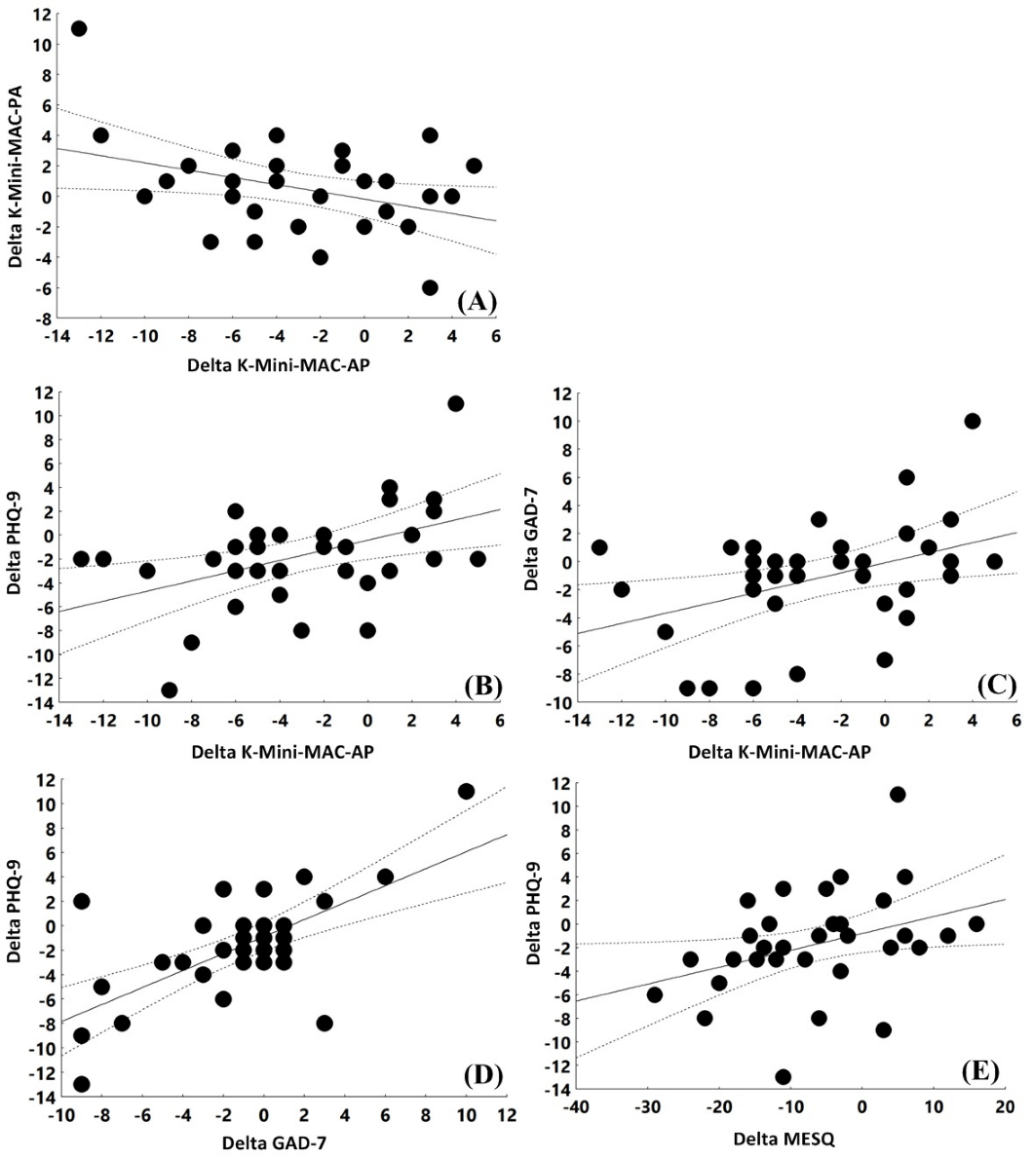
Correlations between score changes for the psychological scales. (A) Changes in the scores for the anxious preoccupation subscale of the Korean version of the Mini-Mental Adjustment to Cancer Scale (K-Mini-MAC-AP) versus those for the positive attitude subscale of the Korean version of the Mini-Mental Adjustment to Cancer Scale (K-Mini-Mac-PA); the statistical results are r=–0.36 and *P*=.04. (B) Changes in the scores for the K-Mini-MAC-AP versus those for the Patient Health Questionnaire-9 (PHQ-9); the statistical results are r=0.46 and *P*=.007. (C) Changes in the scores for the K-Mini-MAC-AP versus those for the Generalized Anxiety Disorder-7 (GAD-7); the statistical results are r=0.41 and *P*=.02. (D) Changes in the scores for PHQ-9 versus those for the GAD-7; the statistical results are r=0.66 and *P*<.001. (E) Changes in the scores for PHQ-9 versus those for the Menopause Emotional Symptoms Questionnaire (MESQ); the statistical results are r=0.35 and *P*=.04.

Changes in the scores for the anxious preoccupation subscale of the K-Mini-MAC Scale were positively correlated with those for the PHQ-9 (*r*=0.46; *P*=.007) and GAD-7 (*r*=0.41; *P*=.02) scores and negatively correlated with those for the positive attitude subscale of the K-Mini-MAC Scale (*r*=–0.36; *P*=.04; Table 4; Figure 4). Changes in the scores for the PHQ-9 were positively correlated with those for the GAD-7 (*r*=0.66; *P*<.001) and MESQ (*r*=0.35; *P*=.04).

**Table 4:** Correlations among score changes for clinical scales within the Cancer Manager group (N=34).

Variables		Δ^a^ CS-SES-K-seeking^b^	Δ WHOQOL-BREF-psy^c^	Δ K-Mini-MAC-AP^d^	Δ K-Mini-MAC-PA^e^	Δ PHQ-9^f^	Δ GAD-7^g^	Δ MESQ^h^
**Δ CS-SES-K-seeking**								
	*r*	1	0.195	0.026	0.042	–0.214	–0.022	–0.111
	*P* value	—^i^	.27	.89	.81	.22	.90	.53
**Δ WHOQOL-BREF-psy**								
	*R*	0.195	1	–0.318	–0.116	–0.039	–0.166	–0.053
	*P* value	.27	—	.07	.51	.83	.35	.77
**Δ K-Mini-MAC-AP Scale**								
	*r*	0.026	–0.318	1	–0.363	0.456	0.406	0.261
	*P* value	.89	.07	—	.04	.007	.02	.14
**Δ K-Mini-MAC-PA Scale**								
	*r*	0.042	–0.116	–0.363	1	–0.111	–0.094	0.008
	*P* value	.81	.51	.04	—	.53	.60	.96
**Δ PHQ-9**								
	*r*	–0.214	–0.039	0.456	–0.111	1	0.656	0.354^*^
	*P* value	.22	.83	.007	.53	—	<.001	.04
**Δ GAD-7**								
	*r*	–0.022	–0.166	0.406	–0.094	0.656	1	0.240
	*P* value	.90	.35	.02	.60	<.001	—	.17
**Δ MESQ**								
	*r*	–0.111	–0.053	0.261	0.008	0.354	0.240	1
	*P* value	.53	.77	.14	.96	.04	.17	—

^a^Δ: the differences between baseline and follow-up assessments.

^b^CS-SES-K-seeking: seeking help and support subscale of the Korean version of the Cancer Survivor Self-Efficacy Scale.

^c^WHOQOL-BREF-psy: psychological health subscale of the World Health Organization Quality of Life Brief Version.

^d^K-Mini-MAC-AP: anxious preoccupation subscale of the Korean version of the Mini-Mental Adjustment to Cancer.

^e^K-Mini-MAC-PA: positive attitude (fatalism and fighting spirit) subscale of the Korean version of the Mini-Mental Adjustment to Cancer.

^f^PHQ-9: Patient Health Questionnaire-9.

^g^GAD-7: Generalized Anxiety Disorder-7.

^h^MESQ: Menopause Emotional Symptoms Questionnaire.

^i^Not applicable.

### User Satisfaction

Regarding the mean scores for all 17 items of the satisfaction survey, they were above an average of 4 points (Table 5), and 26 (76%) out of 34 participants responded with *strongly agree* or *agree* to the question about satisfaction with CAMA use. More than 90% of the participants also indicated the following: CAMA use (32/34, 94%) and the information it provides (33/34, 97%) are easy to understand, CAMA benefits physical health (31/34, 91%) and psychological and emotional health (31/34, 91%) management, the intention to use digital health care apps such as CAMA again (31/34, 91%).

**Table 5:** Cancer Manager (CAMA) user satisfaction survey results (N=34).

Questions	Values, mean (SD)	Values, n (%)^a^
1. Overall, I am satisfied with the use of CAMA.	4.2 (0.9)	26 (76)
2. It is easy to learn how to use CAMA.	4.5 (0.6)	32 (94)
3. The information content introduced by CAMA is helpful.	4.5 (0.7)	30 (88)
4. The information content of CAMA is easy to understand.	4.7 (0.5)	33 (97)
5. The information content of CAMA is interesting.	4.4 (0.8)	30 (88)
6. CAMA helps me manage my physical health.	4.5 (0.7)	31 (91)
7. CAMA helps me understand my cancer treatment process.	4.3 (0.8)	28 (82)
8. CAMA helps me know symptoms caused by cancer.	4.5 (0.7)	29 (85)
9. CAMA helps me control symptoms caused by cancer.	4.4 (0.7)	29 (85)
10. CAMA helps me know side effects of treatment.	4.3 (0.8)	28 (82)
11. CAMA helps me cope with side effects of treatment.	4.3 (0.8)	27 (79)
12. CAMA helps me with my psychological and emotional health.	4.4 (0.7)	31 (91)
13. CAMA helps me understand psychological difficulties related to cancer.	4.4 (0.7)	29 (85)
14. CAMA helps me control psychological difficulties associated with cancer.	4.3 (0.7)	29 (85)
15. CAMA helps me manage my daily life.	4.4 (0.7)	29 (85)
16. I want to use a digital health care app like CAMA again.	4.5 (0.7)	31 (91)
17. I want to introduce CAMA to other patients with breast cancer.	4.5 (0.7)	30 (88)

^a^Percentage of participants who responded with *strongly agree* or *agree* to each question.

As mentioned in the Methods section, we also provided participants with an open-ended item where they could provide feedback on satisfaction and areas for improvement regarding the app, which yielded the following insights. Participants were reportedly satisfied with the ease with which they could access reliable information through the app and appreciated its convenience. They pointed out issues with internet searches, as some answers found through the search engines stemmed from nonexperts and introduced bias. They praised the CAMA app for presenting the opinions of medical professionals, which helped build their trust. Participants emphasized the value of the CAMA app through its provision of trustworthy information and support, as these functions helped reduce their impulsive information-seeking behaviors driven by fear. They mentioned that the collaborative efforts of specialists in hematology, breast surgery, and psychiatry afforded them beneficial, integrated therapeutic information. Some suggested improvements included incorporating patient communication features and consistently updating the platform with more comprehensive and in-depth information.

## Discussion

### Principal Findings

The CAMA app was developed to provide evidence-based, expert-authored, personalized digital information for patients with breast cancer according to their breast cancer type and cancer journey phase. In this study, we explored the potential benefits of the CAMA app, observing patterns that suggest possible efficacy in improving self-efficacy, psychological well-being, and mental adjustment to cancer, although the nonrandomized design limited the robustness of these findings and necessitated cautious interpretation. Compared to the TAU group, those in the CAMA group showed the following: significant increases in their self-efficacy for seeking help and support, psychological well-being, and positive attitude toward cancer; significant reductions in their anxious preoccupation with cancer; and decreased levels of depression, anxiety, and menopausal emotional symptoms. Furthermore, score changes for anxious preoccupation with cancer were positively correlated with those in depression and anxiety and negatively correlated with score changes in positive attitude toward cancer. Notably, score changes in depression were positively correlated with anxiety and menopausal emotional symptoms. The feasibility of the app for the targeted population was generally considered good. These findings offer insights into the app’s positive impact, trust-building outcomes, and opportunities for enhancement (eg, introduction of communication tools and ongoing content enrichment). However, these findings are preliminary and should be validated through future randomized controlled trials.

The CAMA app developed and validated in this study offers a unique and comprehensive approach to breast cancer care, distinguishing it from existing mHealth apps. Unlike many current apps that focus on singular aspects such as symptom management, lifestyle changes, and psychological support [15,49], the CAMA app integrates a wide range of functionalities into a single platform. This includes adherence management, tailored medical information, psychological modules, and logistical features such as scheduling and reminders, addressing the multidisciplinary needs of patients with breast cancer. Furthermore, the CAMA app provides personalized information that dynamically adapts to the user’s breast cancer subtype, ongoing or planned treatments (eg, chemotherapy, surgery, and radiation therapy), and recovery stage. This level of customization ensures that patients receive relevant and actionable content tailored to their specific circumstances, overcoming the limitations of generic mHealth apps, which often fail to address unique patient needs [20,49,50]. In addition, a key innovation of the CAMA app is its *My Care Track* feature, which delivers daily personalized content. This contrasts with many existing apps that require users to actively search for information, making the process time consuming and less engaging [50,51]. By offering concise, context-specific updates, the CAMA app ensures timely support while enhancing user engagement [34,52]. The app’s development was also informed by a multidisciplinary team of health care professionals, including oncologists, surgeons, psychiatrists, and psychologists. Research has highlighted the importance of such collaborative design in creating effective and credible mHealth interventions [53,54]. In addition, the CAMA app addresses usability barriers often cited as challenges in mHealth adoption by simplifying complex medical and psychological information into user-friendly formats and providing interactive tools, such as reminders and visual aids [34,51]. By integrating these features, the CAMA app not only meets the diverse needs of patients with breast cancer but also addresses critical gaps in existing mHealth solutions, particularly the lack of comprehensive, personalized, and evidence-based approaches. Its holistic and patient-centric design demonstrates significant potential to improve both the physical and psychological outcomes of individuals navigating the complexities of breast cancer care [21,54].

In this study, the CAMA group demonstrated significant improvements in self-efficacy for seeking help and support compared to the TAU group after the intervention. The items for this variable included queries about confidence in accessing information, reaching out to individuals for assistance and support, independently handling problems, contacting physicians, and receiving help to address issues [37]. Considering the contents in the items, one can infer that using the CAMA app led participants to experience a heightened sense of confidence in accessing the information they needed whenever necessary, identifying human resources for support (eg, medical professionals), and feeling empowered to tackle challenges independently or with others’ assistance. In a similar context and with another sample of patients with breast cancer, a study applied a web-based cancer management system and found that the score for self-efficacy in coping with cancer-related stress increased significantly over time after system implementation [55]. Moreover, researchers have reported on the self-efficacy–enhancing outcomes of web- or app-based interventions for survivors of breast cancer who completed primary treatment and were undergoing chemotherapy [56,57]. A systematic review and meta-analysis revealed that eHealth-based self-management interventions effectively addressed fatigue and enhanced self-efficacy in adult patients with cancer [58]. Thus, our findings corroborate the crucial role observed in previous research of digital interventions in improving self-efficacy among patients with cancer and survivors of cancer.

The hypothesis established before the study onset was that the scores for the CS-SES-K and its 2 subscales would increase significantly after the intervention. However, the total score and the score for the subscale of managing health problems did not increase significantly. This may be explained by a significant deterioration, possibly caused by factors external to the intervention, in the participants’ objective health status upon the completion of the 12-week intervention compared to the health status at enrollment. Specifically, of the 66 total study participants, some (26/34, 76%) of CAMA group and some (23/32, 72%) were either approaching or currently undergoing chemotherapy at the time of enrollment. At this point, one may infer that the health of the 8 participants of CAMA group and 5 participants of TAU group who were exposed to the side effects of these concoctions of treatments (eg, nausea, fatigue, appetite loss, diarrhea, constipation, skin and nail deformity, and hair loss, particularly during chemotherapy) worsened significantly throughout the study period, which may have influenced their assessments after the intervention. In particular, the managing health problems subscale contains items that query the respondent’s confidence in managing fatigue, physical discomfort or pain, emotional distress, other symptoms, health problems, and performing various tasks and activities. Thus, the deterioration in participants’ physical conditions during and because of cancer treatment may have hindered the potential improvements the intervention could have yielded for the managing health problems subscale. A pilot study showed that an intervention encompassing web-based self-management exercises and diet intervention for survivors of breast cancer improved self-efficacy [59]; however, various studies showed results similar to ours (ie, self-efficacy did not improve). In a previous study conducted in Korea, the level of self-efficacy improved immediately after a 7-week intervention involving education and skill training through telephone counseling for patients with breast cancer. Nonetheless, this effect did not persist and was not observed at the 20-week follow-up [60]. For survivors of incurable cancer using eHealth self-management apps, there was no general enhancement in self-efficacy [61]. Similarly, survivors of cancer experiencing fatigue who were exposed to a web-based intervention aimed at supporting self-management of cancer-related fatigue showed no changes in self-efficacy [49].

The intervention of this study significantly reduced anxious preoccupation and increased positive attitude and mental adjustment to cancer. Mental adjustment to cancer has been defined as an individual’s cognitive and behavioral reactions following a cancer diagnosis [52]. Four types of mental adjustment to cancer have been suggested: (1) helplessness or hopelessness, namely, the tendency to feel overwhelmed and resigned because of cancer and experience feelings of powerlessness and hopelessness; (2) anxious preoccupation, which describes consistent cancer-related anxiety that often leads to the experience of high psychological distress; (3) cognitive avoidance, referring to a tendency to avoid thinking about the condition and its implications and to use distractions as a coping strategy; and (4) positive attitude (fatalism and fighting spirit), describing the adoption of an optimistic perspective toward cancer and the process of seeking growth and meaning in the experience. Even survivors of cancer diagnosed 5 to 6 years ago have been observed to exhibit these specific coping patterns related to their cancer experience [62]. Thus, one’s mental adjustment to cancer encompasses the evaluation of cancer implications (ie, one’s understanding of its effects), subsequent responses (ie, thoughts and actions aimed at minimizing the threat), and involuntary emotional reactions to cancer [52]. Having a fighting spirit seemed to benefit psychological adjustment [39,50,54,63], whereas helplessness or hopelessness and anxious preoccupation consistently correlated with elevated levels of adjustment difficulties or low levels of health-related QOL [51,62]. Overall, the decrease in anxious preoccupation scores and the increase in positive attitude scores after using CAMA suggest potential benefits of the app, aligning with findings from previous research.

Furthermore, a study involving an early, structured psychoeducational group intervention for patients with breast cancer showed a significant reduction in anxious preoccupation after the intervention [64]. Another study on a cognitive existential group therapy intervention for women with early-stage breast cancer demonstrated a trend-level reduction in anxious preoccupation [65]. In a Korean study focused on the effects of an integrated educational program on self-efficacy and coping strategies immediately after a breast cancer diagnosis, significant reductions were observed in anxious preoccupation [66]. However, there is a lack of research on positive attitudes and related improvements following interventions for patients with cancer. The *fatalism* referred to in the subdimension of the K-Mini-MAC Scale has been reported to be more associated with active engagement in spirituality and religious practices rather than passive acceptance of the inevitable [50]. This positive and active form of fatalism is associated with a fighting spirit, a sense of personal control, and patient determination, in which patients take active steps to cure their illness or ameliorate its effects [50]. In this study, changes in anxious preoccupation were negatively correlated with those in positive attitudes. This allows us to assume cautiously that the app reduces anxiety by affording users appropriate psychoeducation along with credible treatment suggestions, which, in turn, increases positive attitudes, including fighting spirit.

Moreover, changes in anxious preoccupation were positively correlated with changes in depression and anxiety. These findings are consistent with evidence depicting fighting spirit as having a consistent correlation with lower levels of anxiety and depression and helplessness, hopelessness, or anxious preoccupation as being more likely associated with depression and anxiety [67,68]. In addition, higher depression levels were correlated with higher anxious preoccupation, helplessness, or hopelessness and lower fighting spirit levels in past literature [69-71].

As discussed in the previous paragraphs, various studies have been devoted to exploring the effect of various types of interventions on the anxious preoccupation of patients with cancer, and they have observed a significant decrease in such preoccupation. However, they seem to have not thoroughly pondered about or explained why there was such a reduction in this single dimension and no reduction in other dimensions of mental adjustment to cancer. Upon careful consideration, we speculated that mental adjustment and coping strategy changes may necessitate cognitive or behavioral shifts, which are more likely to occur over a longer period, requiring a more extended follow-up procedure to be observed. These findings indicate the potential need to complement stress management content intervention strategies with direct intervention strategies, such as coping skills training. For instance, a psychological education program, which included health education, stress management, and coping skills training, was conducted with patients with melanoma immediately after diagnosis, yielding positive results (ie, reduction in passive resignation coping strategies) in the experimental group at the 3-month follow-up [72].

The CAMA intervention also significantly reduced depression, anxiety, and menopausal emotional symptom levels, with changes in depression levels being significantly correlated with those in anxiety and menopausal emotional symptoms. Individuals diagnosed with breast cancer often face elevated psychological challenges (eg, feelings of anxiety and depression) that can adversely influence their QOL and chances of survival [73,74]. Accordingly, scholars have recognized the crucial role of psychological support in breast cancer treatment and established various related interventions [75], including psychoeducational interventions that encompass training to handle emotions, receive information, express feelings, discuss concerns, solve problems, receive support, and develop coping strategies [76]. A systematic review revealed that psychoeducational interventions significantly reduced anxiety and depressive symptoms among women diagnosed with breast cancer [75]. Another systematic review and meta-analysis study focused on internet-based psychoeducational interventions further highlighted a significant effect on decreasing depression and fatigue in patients with cancer [77]. Following these pieces of evidence, we have incorporated breast cancer–related medical and surgical treatment information and an equivalent amount of psychoeducational information into the CAMA app’s content, including the following: introductory information on the psychological responses patients may experience during different treatment stages, coping strategies, adaptive or maladaptive symptoms, behavioral therapies, potential psychological reactions of patients and caregivers, and psychological information caregivers should be aware of. These different pieces of information were included based on the belief that they would exert alleviating effects on patients’ depression and anxiety.

In this study, the CAMA group (vs the TAU group) demonstrated significant improvements in psychological well-being. Previous studies’ results on the impact of psychoeducational interventions on the QOL of patients with cancer are consistent with those of this study. For instance, a meta-analysis demonstrated that internet-based psychoeducational interventions significantly decreased depression and fatigue and the insufficiency of evidence to support their effects on psychological distress and QOL in individuals with cancer [77]. A recent systematic review of studies conducted with survivors of breast cancer suggested that psychoeducation significantly improved QOL and alleviated anxiety symptoms but not depression [73]. Our study did not show significant results for any QOL subscale other than psychological well-being; we speculate that the nature of the items within the WHOQOL-BREF may have contributed to these results or their lack thereof. For instance, items within the physical health subscale (eg, energy levels, physical functioning, sleep, and occupational concerns) encompass variables heavily influenced by the medical cancer treatment process (ie, chemotherapy, surgery, or radiation therapy). Thus, similar to the reasoning presented earlier for the nonsignificant changes regarding self-efficacy (as measured by the CS-SES-K), the deterioration in participants’ actual physical conditions caused by cancer treatment may have hindered the intervention’s influence on QOL.

The findings of this study can be interpreted through the lens of the CCM and the HBM, both of which were key to the development of the CAMA app. The CCM emphasizes the importance of providing ongoing, coordinated, and patient-centered care, particularly when managing chronic diseases such as breast cancer [24,25]. In line with this model, the CAMA app facilitated continuous support and empowered patients to take an active role in their health management by integrating medical, psychological, and social resources. This was reflected in the significant improvements observed in self-efficacy, particularly in seeking help and support, and in the reduction of psychological distress among users. Analyzing these findings from the perspective of the HBM further enriches the discussion, as this model focuses on the cognitive and emotional factors that influence health behaviors [29]. The CAMA app was designed to enhance patients’ perceived control over their health by providing reliable information, managing treatment schedules, and offering psychological support. At this point, considering the reduction in anxiety, depression, and menopausal emotional symptoms observed in the CAMA group, we suggest that our findings align with the HBM’s premise, namely, that addressing perceived barriers to and emphasizing the benefits of proactive health behaviors can lead to better psychological outcomes [31,78,79]. The app’s success in fostering a positive attitude and reducing anxious preoccupation among patients with breast cancer suggests that it effectively addressed users’ concerns and motivated them to engage in healthier behaviors. These suggestions align with the principles of both the CCM and HBM.

### Limitations

This study had some limitations. First, it used a nonrandomized design, chosen for pragmatic reasons such as feasibility and logistical constraints. While this facilitated implementation in a real-world clinical setting, it also introduced limitations, including potential selection bias and reduced generalizability. Baseline comparisons between the intervention and control groups showed no significant differences, reducing the need for adjustments in analyses. However, the intervention group included participants willing to use the app and with access to compatible smartphones, which may have influenced the outcomes. Thus, differences between groups could partly reflect these pre-existing characteristics rather than the app intervention alone. As the lack of randomization prevents the exclusion of confounding variables, the findings should be interpreted as exploratory with caution. Future randomized controlled trials are necessary to confirm these preliminary results. Second, the sample size was small, and the follow-up had a short duration. However, considering the nature of this feasibility study, its aim of rapidly assessing the intervention’s usability, and providing empirical evidence for better-designed future research, these limitations can be deemed acceptable. Furthermore, the power calculations demonstrated that the sample size, albeit small, was sufficient for effect validation. Third, this study included participants with significant divergences regarding the time since breast cancer diagnosis (ie, ranging from a few months to 20 years), potentially limiting the findings owing to variations in cancer experiences. Cancer-related self-efficacy and adjustment styles tend to change throughout the cancer journey as they are influenced by condition controllability [80]. Thus, greater sample homogeneity could lead to differential results, implying that future long-term randomized controlled trials must use larger samples and conduct subgroup analyses based on patients’ medical characteristics, cancer journey phase, and treatment types. Fourth, this study did not directly assess physical health and health care use–related indicators. Future researchers should consider direct physical health indicators and health care use–related indicators, such as the following: blood parameters, adverse events and postoperative complications, treatment adherence (medication and outpatient visits), screening adherence (cancer-related and routine checkups), health care costs, or unplanned emergency department and outpatient visits. Finally, app effectiveness was assessed using only subjective measures. Future researchers should collect and analyze data on variables such as medication adherence, compliance with outpatient visits, and objectively measurable health indicators (eg, heart rate variability and blood parameters) to evaluate app efficacy more comprehensively.

### Conclusions

The mobile app for assisting in the self-management of patients with breast cancer, CAMA, was deemed feasible and shows potential for enhancing the patients’ self-efficacy regarding seeking help and support, positive attitude toward cancer, and psychological well-being. In addition, its use may help reduce their anxious preoccupation with cancer, depressive mood, anxiety, and menopausal emotional symptoms. However, the nonrandomized study design highlights the need for further randomized controlled trials to confirm these preliminary findings.

## Appendix

Multimedia Appendix 1 TREND (Transparent Reporting of Evaluations with Nonrandomized Designs) checklist.

## Abbreviations

CAMA: Cancer Manager
CCM: chronic care model
CS-SES-K: Korean version of the Cancer Survivor Self-Efficacy Scale
GAD-7: Generalized Anxiety Disorder-7
HBM: health belief model
K-Mini-MAC: Korean version of the Mini-Mental Adjustment to Cancer
MESQ: Menopause Emotional Symptoms Questionnaire
mHealth: mobile health
PHQ-9: Patient Health Questionnaire-9
Q and A: question and answer
QOL: quality of life
TAU: treatment as usual
TREND: Transparent Reporting of Evaluations with Nonrandomized Designs
WHOQOL-BREF: World Health Organization Quality of Life Brief Version
